# Assessment of soy phytoestrogens' effects on bone turnover indicators in menopausal women with osteopenia in Iran: a before and after clinical trial

**DOI:** 10.1186/1475-2891-4-30

**Published:** 2005-10-29

**Authors:** Arezoo Haghighian Roudsari, Farideh Tahbaz, Arash Hossein-Nezhad, Bahram Arjmandi, Bagher Larijani, Seyed Masoud Kimiagar

**Affiliations:** 1 School of Nutrition and Food Technology, Shaheed Beheshti University of Medical Sciences, Tehran, Iran; 2 Endocrinology & Metabolism Research center, Tehran university of Medical science, Tehran, Iran; 3 Department of Nutritional Sciences, Oklahoma State University, Stillwater, OK, USA

**Keywords:** soy protein, Isoflavones, postmenopausal women, bone metabolism markers, osteopenia

## Abstract

**Background:**

Osteoporosis is the gradual declining in bone mass with age, leading to increased bone fragility and fractures. Fractures in hip and spine are known to be the most important complication of the disease which leads in the annual mortality rate of 20% and serious morbidity rate of 50%. Menopause is one of the most common risk factors of osteoporosis. After menopause, sex hormone deficiency is associated with increased remodeling rate and negative bone balance, leading to accelerated bone loss and micro-architectural defects, resulting into increased bone fragility.

Compounds with estrogen-like biological activity similar to "Isoflavones" present in plants especially soy, may reduce bone loss in postmenopausal women as they are similar in structure to estrogens.

This research, therefore, was carried out to study the effects of Iranian soy protein on biochemical indicators of bone metabolism in osteopenic menopausal women.

**Materials and methods:**

This clinical trial of before-after type was carried out on 15 women 45–64 years of age. Subjects were given 35 g soy protein per day for 12 weeks. Blood and urine sampling, anthropometric measurement and 48-h-dietary recalls were carried out at zero, 6 and 12 weeks. Food consumption data were analyzed using Food Proccessor Software. For the study of bone metabolism indicators and changes in anthropometric data as well as dietary intake, and repeated analyses were employed.

**Results:**

Comparison of weight, BMI, physical activity, energy intake and other intervening nutrients did not reveal any significant changes during different stages of the study. Soy protein consumption resulted in a significant reduction in the urinary deoxypyridinoline and increasing of total alkaline phosphatase (p < 0.05), although the alterations in osteocalcin, c-telopeptide, IGFBP3 and type I collagen telopeptide were not significant.

**Conclusion:**

In view of beneficial effect of soy protein on bone metabolism indicators, inclusion of this relatively inexpensive food in the daily diet of menopausal women, will probably delay bone resorption, thereby preventing osteoporosis.

## Backgrounds

Menopause is a period normally occupying one-third of women's life [1]. Reduced bone density is one of the most prominent symptoms during menopause [2].

Osteoporosis is a serious problem for postmenopausal women which increases the risk of bone fracture and worsens with age, increasing from 4% in 50–59 year age bracket to 50% in 80 years old women. Bone fractures are also prevalent in these women [3]. Today estrogen therapy (ERT) and drugs like bisphosphonates, calcitonin and raloxifene is employed to prevent and treat osteoporosis [4]. However, side effects such as breast cancer, endometrial adenocarcinoma [5] have limited the acceptance of these medications among women [6] and only 35% to 40% of women ever start ERT, and many do not continue it [7].

Epidemiologic studies have shown osteoporotic fractures, cardiovascular disease, postmenopausal symptoms and some cancers to be less prevalent in Asians compared to their western counterparts. Hip fracture, for example, is 50–60% less frequent among Asian compared to western women [8]. This advantage is gradually anihilated as Asian adapt western lifestyle [9]. These observations, prompted researchers to scrutinize Asian dietary habits. Soy is a part of Asian traditional diet [10], showing some relationship with the above-mentioned diseases [9].

Estrogen-like compounds such as isoflavones existing in plant foods specially soy [11,12] can curb reduced bone density in menopausal women, due to their structural similarity [13]. Some studies have not, however, supported clearly the role of soy isoflavones in preventing osteoporosis [14].

Isoflavones are phyto-estrogens similar to women's estrogens and are bound to cellular estrogen receptors in various organs, thus phytoestrogens affinity is weak compared to human's estrogens. Recent studies have shown that cells have two types estrogen receptors α and β. Human estrogens have more affinity to α-receptors, whereas, isoflavones have high affinity to β-receptors. β-receptors exist in brain, bone, bladder and vascular epithelium, being important in the function of non-steroid estrogens [15].

As soy cultivated in Iran is of different variety, namely "Gorgan" compared to other studies and because the Iranian food habits and food pattern is different [16] which might affect the metabolism of nutrients and isoflavones, this study was conducted to assess its effects compared to other countries. Furthermore there are still contradiction, in the literature regarding the role of soy isoflavones which justify implementation of this study.

## Methods

This clinical trial of before and after type was carried out in 2003. Women referring to the osteoporosis clinic of Endocrinology & Metabolism Research Centre of Tehran University of Medical Sciences for bone density measurement were screened to find osteopenic subjects and 15 postmenopausal 45–64 year old women were selected. Those women between 1 to 10 years postmenopause who were non-smokers and free from diseases entered this study.

Information on weight, height, body mass index, two 24-hr food consumption recall and physical activity were collected at the start, 6 and 12 weeks of the study. Soy protein at 35 g level containing 98.3 mg isoflavones were given to subjects daily. Subjects were provided with a special cup for measuring soy. Cooking instructions were also given to the subjects.

Blood and urine samplings were done in 3 stages, in the beginning and at the end of 6^th ^and 12^th ^week. Blood and urine samples were kept frozen until the end of the twelfth week at -80°C. Serum biochemical indicators were measured on the same day for all samples. Total alkaline phosphatase was assayed calorimetrically with Hitachi 902 autoanalyzer, osteocalcin by IRMA method using Biosource kit and Wizard gamacounter, IGFBP3 and c-telopeptide by ELISA using Biosources and Bioscience diagnostics respectively. Type I collagen telopeptide was determined by RIA method using Orion-Diagnostica on Wizard gama counter and urinary creatinine by colorimetrically method [17]. Food Processor software was used for food consumption survey and SPSS (version 11.5) was employed for statistical analysis of the data. All quantitative variables were then examined by Kolmogrof-Smirnof (KS) to ensure normality of distribution. To analyze any possible changes in food intake, intervening and biochemical variables in 3 stages, repeated measurement analysis was utilized. The purpose of this analysis was to ensure lack of significant changes of the variables. Significance level was set at below 5 percent (P < 0.05).

## Results

Subjects' anthropometric data are shown in Table 1. Mean age was 52.9 ± 4.3 years, years post menopause 5.47 ± 3.4 years and mean height 157.4 ± 7.2 centimeters. Mean body mass index and physical activity level remained unchanged. Mean food consumption figures were not different at 6 and 12 weeks compared to the start of the study (Table 2). Mean bone metabolic indicators for the 3 stages are given in Table 3. After 12 weeks of soy consumption total serum alkaline phosphatase (TALP) significantly increased while urinary deoxypyridinoline (DPD) decreased (P < 0.05). Other indicators namely osteocalcin, insulin growth factor binding protein (IGFBP3), c-telopeptide and type-I collagen telopeptides did not change significantly.

**Table 1:** Subjects anthropometric data in 3 stages of the study (n = 15).

**Indicators**	**At the start**	**6 weeks**	**12 weeks**
**Weight (kg)**	68 ± 7.5*	68 ± 7.6	68 ± 7.8
**BMI (kg/m^2^)**	27.4 ± 3**	27.4 ± 3	27.4 ± 3

* Mean ± SD

** Mean height was 157.2 ± 7.2.

**Table 2:** Subjects mean food intake in 3 stages of the study (n = 15).

**Variables**	**at the start**	**6 weeks**	**12 weeks**	P value**
**Energy (kcal)**	1933.4 ± 302.5*	2033.2 ± 420.3	1902.8 ± 308.6	NS***
**Protein (g)**	74.6 ± 12.3	71.9 ± 17.4	74.8 ± 10.2	**NS**
**Calcium (mg)**	999.3 ± 460.2	966.8 ± 443.7	1014 ± 436	**NS**
**Phosphore (mg)**	873.2 ± 228.9	853.1 ± 273.9	866.4 ± 200.6	**NS**

* Mean ± SD

** significant level was set at below 5 percent (P < 0.05).

*** To analyze changes in 3 stages, repeated measurement analysis was utilized.

**Table 3:** Bone metabolism biomarkers in 3 stages of the study (n = 15).

Variables*	**At the start**	**6 weeks**	**12 weeks**	**P value**^†^
**TALP **(IU/l)	237.5 ± 85.4**	300.4 ± 294.1	281.3 ± 80.5	**<0.05**
**OC **(ng/ml)	11.4 ± 4.8	12.7 ± 5.7	11.5 ± 4.8	**NS**^††^
**IGFBP3 **(ng/ml)	3304.6 ± 728.6	3221.6 ± 534.4	3103.9 ± 639.8	**NS**
**DPD **(nmol/mmol)	7 ± 1.2	5.9 ± 1.1	5.1 ± 2.1	**<0.05**
**C-TX **(ng/ml)	0.79 ± 0.49	0.8 ± 0.4	0.8 ± 0.3	**NS**
**ITCP **(μg/l)	4.6 ± 0.9	4.3 ± 0.9	5.9 ± 1.1	**NS**

* TALP: Total alkaline phosphatase (IU/l); OC: Osteocalcin (ng/ml); IGFBP3: Insulin like growth factor binding protein 3 (ng/ml); DPD: Deoxypyridinoline (nmol/mmol); C-TX: C-Telopeptides (ng/ml); ITCP: Carboxy terminal telopeptides of type I collagen (μg/l)

** Mean ± SD

† To analyze changes in 3 stages, repeated measurement analysis was utilized.

†† significant level was set at below 5 percent (P < 0.05).

## Discussion

The results demonstrated soy protein consumption to have caused increase in TALP and reduction in DPD in menopausal women with osteopenia, while other parameters were not significantly difference. Comprehensive human studies on the effect of soy on TALP have not been carried out. In Arjmandi et al studies on rats a slight but insignificant increase in TALP was seen [18-20]. The crucial role of gut microflora in the metabolism of isoflavones in human beings has also been previously explored [21]. In vivo studies proved that bacteria in the gastrointestinal tract play an important role in determining the magnitude and pattern of isoflavone bioavailability [22]. However, only 30 to 40% of the population can produce equol from daidzein and interindividual differences in the bacteria responsible for equol production [23,24]. Register et al observed a significant fall in TALP in monkeys after 12 weeks [25]. Animal models such as monkey may convert daidzein into equol more readily than 30 to 50% of humans. It has been shown that equol possesses more estrogen-like properties than daidzein. This is why isoflavone efficacy has been less pronounced in monkeys and the results have been reported as reduced TALP levels [26,27].

TALP is an insensitive marker for bone formation compared to bone-specific alkaline phosphatase (BAP) or osteocalcin (OC). In this study, serum osteocalcin as a sensitive marker for bone formation has not changed during interventional period, indicating that soy protein may not enhance bone formation.

With regard to bone resorption, our results showed reduced urinary DPD levels following soy consumption which agrees with the finding of other investigators [28-30]. The effect of isoflavones on this indicator is so strong that Uesagi et al [31] observed consuming 61.8 mg of isoflavone for 4 weeks results in a significant reduction in urinary DPD. It can be said that DPD acts as a bridge between collagen fibrils which enter urine with collagen breakdown. As this is a very specific marker for bone resorption, its significant reduction in our study suggests soy consumption may prevent degradation of collagen the major protein in bone matrix [17].

Other serum indicators of bone metabolism were not affected in our study. In most studies on the effects of isoflavones in rats these phytochemicals, have been reported to have caused rises [32], no change [19,20] and even reduction [14] in bone formation as well as reduction or no change [33,34] on bone resorption. The changes observed in this study, therefore, are not contradictory to other studies and slight differences observed may be attributed to sample size, isoflavone dosing, period of intervention and dissimilarity of the studied groups.

## Conclusion

In conclusion it seems soy protein can be effective in protecting bone mass through curbing bone resorption specially in high risk groups as was demonstrated in our osteopenic subjects but not enhance bone formation. Different studies have reported intake of 70–90 milligrams of isoflavones per day to be effective. Soy protein in our study provided 98 mg of isoflavones which is in accordance with other studies [35,36]. Some have reported lesser amounts can be effective in longer periods of time [37]. Soy protein consumption, thus, is a valuable plant estrogen which can be recommended for osteoporosis prevention.

## List of abbreviations

**BAP**: Bone-specific Alkaline Phosphatase

**BMI**: Body Mass Index

**CTX**: Collagen type I cross-Linked C-telopeptide

**Dpd**: Deoxypyridinoline

**ELISA**: Enzyme Linked Immunosorbent assay

**IU/l**: International Unit/liter

**IGFBP3**: Insulin Like Growth Factor-Binding Protein 3

**IRMA**: Immuno Radiometric Assay

**ITCP**: Serum Carboxyterminal telopeptide of type I collagen

**Kcal**: Kilocalorie

**Kg/m**^2^: kilogram/meter^2^

**Ks**: Kolmogrof-smirnof

**mg**: miligram

**n mol/m mol**: nano mol/mili mol

**ng/ml**: nanogram/mililiter

**Oc**: Osteocalcin

**RIA**: Radioimmunoassay

**TALP**: Total Alkaline Phosphatase

**μg/L**: microgram/Liter

## Competing interests

The author(s) declare that they have no competing interests.

## Authors' contributions

This research is the title of **AHR**'s thesis. She has designed the frame of research and performed the subjects' selection, intervention, follow up of subjects, data gathering and data analysis and finally writing and editing of this manuscript was carried out by her.

**FT**: Advisor of thesis.

**AHN **assisted with subjects collection and data analysis. He also contributed in performing the laboratory tests.

**BA **Measured the soy phytoestrogens.

**BL and MK**: conceived of the study and participated in its design.
